# Functional magnetic resonance imaging based on Chinese tasks to protect language function in epileptics

**DOI:** 10.1002/brb3.1979

**Published:** 2020-12-30

**Authors:** Peng Wang, Feizhou Du, Jianhao Li, Hongmei Yu, Chencheng Tang, Rui Jiang

**Affiliations:** ^1^ Department of Radiology The General Hospital of Western Theater Command Chengdu China

**Keywords:** brain language function, epilepsy, functional magnetic resonance, intraoperative EEG

## Abstract

**Objective:**

To evaluate the efficacy of functional magnetic resonance imaging (fMRI) based on Chinese tasks to protect the language function in epileptics.

**Materials and Methods:**

A total of 34 native Chinese patients with epilepsy were enrolled and examined with BOLD‐fMRI scan based on six Chinese tasks. The epileptics were randomly divided into the control group (*n* = 15) and the experimental group (*n* = 19). The control group underwent the hollowing and multiple subpial transection operation only based on intraoperative EEG, while the experimental group was under notification of task‐state fMRI results in addition. Whereafter, the language ability of patients was evaluated by ABC assessment.

**Results:**

The brain regions related to Chinese function activated by different tasks were remarkably distinct and mainly concentrated in the temporal lobe and frontal lobe. In ontoanalysis, the activation signals of the fusiform gyrus, parahippocampal gyrus, hippocampus, and precentral gyrus were generally low or even could not be detected. Unlike ontoanalysis, group analysis showed that the main effect regions of AN and PN task were in right superior temporal gyrus. The main effect regions of FF and VFC task were in right middle temporal gyrus. The main effect region of SF task was in left superior temporal gyrus. The main effect region of VFL task was in right middle frontal gyrus. The ABC assessment score of the control group 6 months after surgery was significantly lower than that 1 week before surgery (*p* < .05), while there was no significant difference in the experimental group, and the score of the experimental group was higher than that of the control group.

**Conclusion:**

In the surgical treatment of epilepsy, a personalized surgical plan, based on task‐state fMRI and intraoperative EEG, can be developed according to the difference of activation areas to protect the language function and improve the quality of life in postoperative patients.

## Graphical abstract

A total of 34 native Chinese epileptics were enrolled and examined with BOLD‐fMRI scan based on six Chinese tasks. The epileptics were divided into the control group (*n* = 15) and the experimental group (*n* = 19). The control group underwent the hollowing and multiple subpial transection operation only based on intraoperative EEG, while the experimental group was under notification of task‐state fMRI results in addition. Whereafter, the language ability of patients was evaluated by ABC assessment. The results showed the activated brain regions could be detected by task‐state fMRI, which were mainly concentrated in temporal lobe and frontal lobe, and the language function of patients was better protected after operation. In the surgical treatment of epilepsy, a personalized surgical plan, based on Chinese language task‐state fMRI and intraoperative EEG, can be developed according to the difference of activation areas to protect the language function and improve the quality of life in postoperative patients.

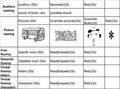

## 1 INTRODUCTION

Task‐state magnetic resonance imaging (fMRI) can design language task form in different aspects to observe the activated brain functional region and localize the position of brain function region related to language processes (Kircher et al., 2011). On the one hand, minimally invasive surgery based on electroencephalography (EEG) localization is not enough to minimize the risk of brain function damage caused by surgery (Boling, 2018; Smith, 2005; Téllez‐Zenteno et al., 2005). On the other hand, language functional areas of brain are very hard to be identified in Chinese patients with epilepsy. Because Chinese has its particularity requiring more brain regions to participate and is different in pronunciation, intonation, grammar, vocabulary, and expressions compared with Western languages (Yang et al., 2015). Chinese characters are a symbol system and form an ideographic syllabic logographic language, which is dominated by vowels without a consonant cluster, and their tones have different meanings. And the main word‐formation method of Chinese characters is word root of recombination, their important feature of grammar is the word order and functional word (Ding et al., 2015; Lu et al., 2007; Xia & Andrews, 2014). What's more, there is no simple corresponding relationship between word class and grammar of Chinese, and the combination of the words is subject to the semantic context. Due to individual differences, there are differences in language functional areas between individuals. Besides, pathological damage of epilepsy will lead to the redistribution of language network connections (Sidhu et al., 2016), and the division of Broca's and Wernicke's dominant language areas is too rough (Linehan et al., 2011). Hence, a more personalized localization of language function is urgently needed to achieve preoperative damage assessment and intraoperative protection of language function, and task‐state fMRI research with Chinese characteristics is very valuable.

## 2 MATERIALS AND METHODS

### 2.1 Participants

This study was approved by the Ethical Committee of The General Hospital of Western Theater Command. The general information of 34 patients with epilepsy was shown in Table 1. The inclusion criteria were as follows: (a) epileptic seizures were diagnosed as the result of abnormal discharge of neurons based on the video of EGG, (b) the native language of patients was Chinese, and the patients were able to recognize Chinese characters, and read Pinyin, (c) patients had no history of mental illnesses and drug abuse, (d) patients had no history of craniocerebral trauma and operation, (e) patients had no history of stroke, encephalorrhagia and intracranial infection, and (f) patients and their families understood and agreed with the purpose of the study, and signed the informed consent for examination. The exclusion criteria were as follows: (a) patients whose task‐state image scanning failed or the data did not meet the analysis requirements and (b) patients or their families didn't agree to carry on this study.

**TABLE 1:** The general information of 34 patients

Group	Patient number	Age	Gender	Handedness	Handedness score
		(yr)			
The control group	1	41	F	R	80
	2	27	F	R	73
	3	27	M	B	61
	4	21	F	R	85
	5	24	F	R	81
	6	35	F	R	76
	7	31	F	R	75
	8	35	F	R	51
	9	25	F	R	62
	10	24	F	R	83
	11	24	M	R	42
	12	29	M	R	76
	13	28	M	R	81
	14	30	F	R	58
	15	48	M	R	62
The experimental group	16	33	F	R	83
	17	27	M	R	83
	18	36	F	R	58
	19	39	F	R	83
	20	39	M	R	75
	21	28	M	R	50
	22	20	M	R	58
	23	20	M	R	50
	24	21	F	R	58
	25	19	F	R	50
	26	19	F	B	20
	27	41	M	R	91
	28	59	F	R	83
	29	25	M	R	100
	30	19	F	L	75
	31	44	F	B	33
	32	20	F	B	−8
	33	18	F	B	−8
	34	35	M	R	75

Note

In Gender, F, female, M, male; in Handedness, R, right hand, L, left hand, B, bilateral hands. Handedness score was evaluated by the Edinburgh hand preference inventory (Oldfield, 1971).

### 2.2 ABC assessment

The Aphasia Battery of Chinese (ABC) assessment, which was established by the Neuropsychology Department of the First Affiliated Hospital of Beijing Medical University (Gao et al., 1992), was used to quantitatively assess the language ability of patients, including verbal fluency, repetition, comprehension, naming, reading, and writing. The total score is 750. The language ability of patients was evaluated 1 week before surgery and 6 months after surgery with different versions of the battery of tests to avoid test–retest effects.

### 2.3 Language task design

**FIGURE 1:**
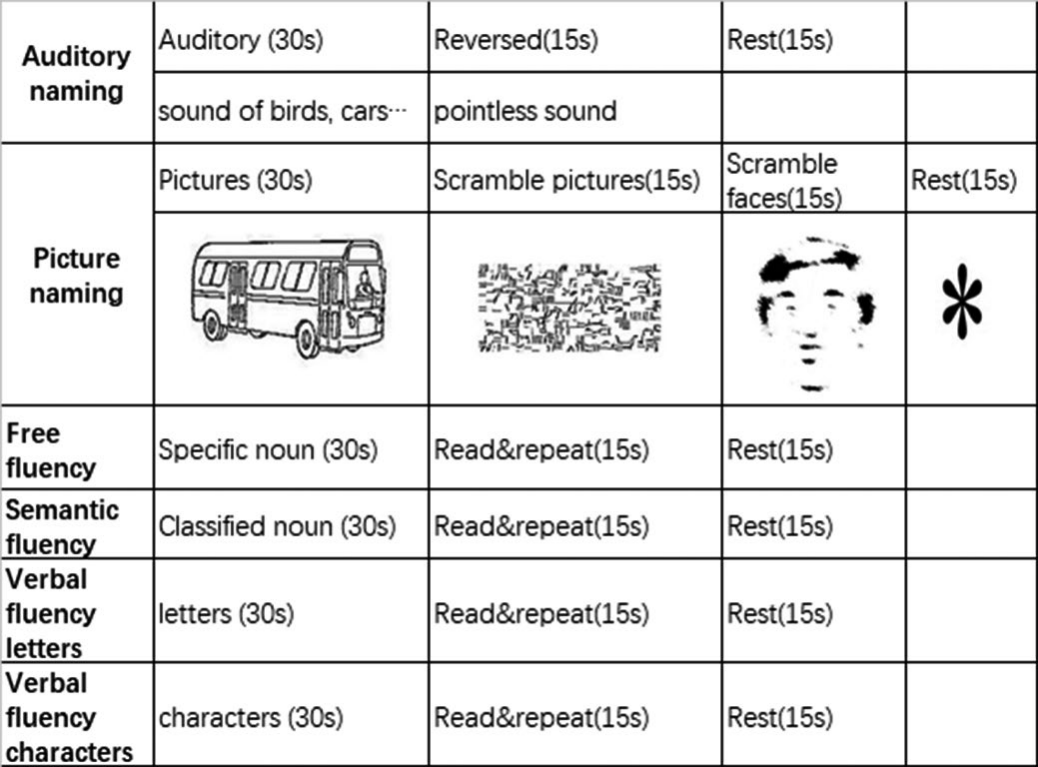
Language paradigms. All paradigms utilized a blocked experimental design. Six experimental tasks were employed

All tasks were arranged by the professional stimulus task design program E‐prime based on previous studies (Ci et al., 2016; Pauselli et al., 2018; Wang et al., 2016) and displayed onto the screen via a projector. Language stimulus software was Visual&Audio Stimulation System for fMRI developed by Sinorad medical company. The experiment was planned to use block design, with stimulus (Task) and rest alternating. According to the characteristics of Chinese and the experimental elements of task‐mode, six experimental modules were designed as follows and shown as Figure 1. All participants watched pictures through a small mirror on the head coil for visual stimulation, heard the voice through a magnetic resonance compatible speech system for auditory stimulation.

#### 2.3.1 Auditory naming (AN)

There were five cycles in AN. Each cycle included a 30 s stimulus task and two control tasks, namely the reverse voice (Reversed, Re) and "*" for 15 s, respectively. During the stimulus task, participants were asked to name the items based on description they had heard. For example, stimulus task was "quacking", whose answer should be "duck". During retask, participants only could hear the voice, but not know voice content, and were required to number "1, 2." When "*" appeared on the screen, rest started.

#### 2.3.2 Picture naming (PN)

There were five cycles in PN, and each cycle consisted of four modules. Line‐like black‐and‐white pictures, which were common objects or animals and plants on white background (Snodgrass atlas, provided by the institute of psychology, Chinese Academy of Sciences), were displayed for 30 s. Participants were asked to name the items in the picture. Scrambled faces (SF) pictures were displayed for 15 s on the screen, scrambled pictures were displayed for 15 s, and rest pictures "*" were displayed for 15 s. Participants were asked to number "1, 2" over Scrambled faces (SF) pictures and scrambled pictures and rested when saw “*”.

#### 2.3.3 Free fluency (FF)

There were five cycles in FF, and each cycle consisted of 3 modules. During stimulus task, Chinese characters were displayed for 30 s on the screen, and participants were asked to list as many words associated with the Chinese characters as possible until the next task began. For example, Chinese characters "glass" could be associated with "Windows, houses, furniture… ". In the next 15 s, participants were asked to just read and repeat (RR) the words that appeared on the screen, but not to think. For example, "science" was read and repeated once by participants. When "*" appeared, participants rested.

#### 2.3.4 Semantic fluency (SF)

There were five cycles in SF, and each cycle consisted of 3 modules. During the first 30 s of stimulus task, participants were asked to list as many words belonging to the Chinese characters on the screen as they can. For example, the word "fruit" could be listed as "apple, banana, peach… ". In the next 15 s, participants were asked to read and repeat (RR) the words appeared on the screen, such as "insect". When "*" finally appeared, participants rested.

#### 2.3.5 Verbal fluency letter (VFL)

There were five cycles in VFL, and each cycle consisted of 3 modules. According to the given initials, the initials and different finals could be matched to form Pinyin during the first 30 s of stimulus task. For example, initials "P" could be divided into "pa, po, pin…". In the next 15 s, participants were asked to read and repeat (RR) the words appeared on the screen. When "*" appeared, participants rested.

#### 2.3.6 Verbal fluency characters (VFC)

There were five cycles in VFC, and each cycle consisted of 3 modules. According to the given Chinese characters, participants were asked to combine words with the meaning of the Chinese character during the first 30 s of stimulus task. For example, the Chinese character "water" could be grouped as "flood, sewage, boiling water…". In the next 15 s, participants were asked to read and repeat (RR). When "*" appeared, rest started.

### 2.4 MRI scan

All the participants were informed about the significance of the experiment design to strive for positive coordination and trained to keep their heads still while performing the above tasks. Before examination, the participants also rested quietly to adapt to the MR scanning environment.

All data were acquired using a 3.0 T Philips NMR with an 8‐channel array head coil for reception and the body coil for transmission. The blood oxygenation level‐dependent (BOLD) signals collection was performed with single‐shot echo‐planar imaging(EPI), and scanning parameters were TR = 3,500 ms, TE = 60 ms, N.E.X = 1, FLIP Angle = 90°, Matrix = 128 × 128, FOV = 24 cm × 24 cm, Thickness = 6 mm, Gap = 1 mm. The whole‐brain scan was 21 layers, and the parameters of transverse T_1_WI (SE sequence) were TR = 500 ms, TE = 12 ms, N.E.X = 1, FOV = 24 cm × 24 cm, FLIP angle = 90°, Matrix = 192 × 256, Thickness = 6 mm, Gap = 1 mm. The parameters of Gradient‐echo T2 were TE = 25 ms, TR = 2000 ms, Thickness = 2.5 mm, FOV = 24 cm, Matrix = 64×64, Pixel = 3.75 × 3.75 mm, which were used for fMRI acquisition. FOV covered frontal lobe and temporal lobe to the maximum extent, and the signal‐to‐noise ratio of frontotemporal lobe image also was increased.

### 2.5 fMRI data analysis

The data were analyzed using SPM8 software (Wellcome Department of Cognitive Neurology, http://www.fil.ion.ucl.ac.uk/spm/), with the steps as follows: (a) Realign: 3D sinc interpolation algorithm was applied to register all functional images with the first phase functional images, then correct the head movement, and acquire the corrected image and average image. (b) Normalize: a scanner specific template was created from 30 healthy volunteers, 15 patients with left hippocampal sclerosis, and 15 patients with right hippocampal sclerosis. With montreal neurological institute (MNI) standard brain template as the reference, standardized parameters of functional images could be acquired by nonrigid body transformation, which was used to normalize all functional images. (c) Spatial smoothing of functional images: the normalized functional images were spatially smoothed with a Gaussian kernel of 6 mm FWHM.

After the convolution of the hemodynamic response function (HRF), the stimulation mode function was established as a design matrix. The positions of language functional areas activated by different language stimulus task were recorded, and the parameters of active regions, such as strength and size, were measured at the same time. The general linear model (GLM) was applied to estimate the parameters of the time series of functional images, and the activation level was defined as *p* < .001 after correct inspection. For intergroup analysis, different tasks were analyzed by 2nd level single sample *t* test installed in statistic parametric mapping (SPM8). All data in the same group were calculated and determined as activation, and the activation level was defined as *p* < .001 after correct inspection. The activation region of language task was more than 10 voxels, otherwise, and it was identified as spike and excluded.

### 2.6 Surgery treatment

**FIGURE 2:**
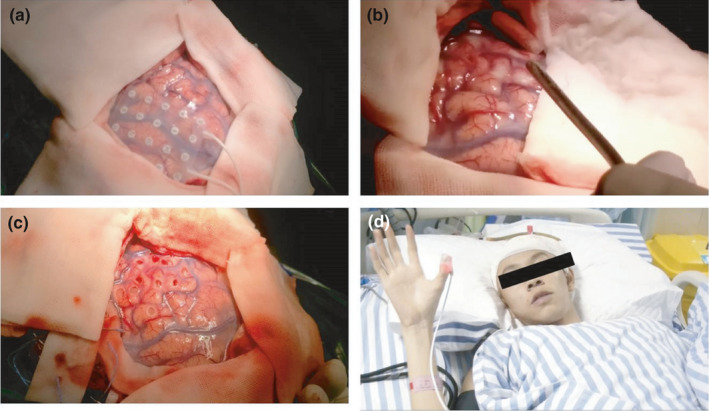
An 18‐year‐old patient with recurrent limb stiffness and sustained ineffective drug treatment for 1 year. (a) EEG marking the seizers in the operation; (b) making the "hollows"; (c) the "hollows"; (d) Good recovery after the operation

**FIGURE 3:**
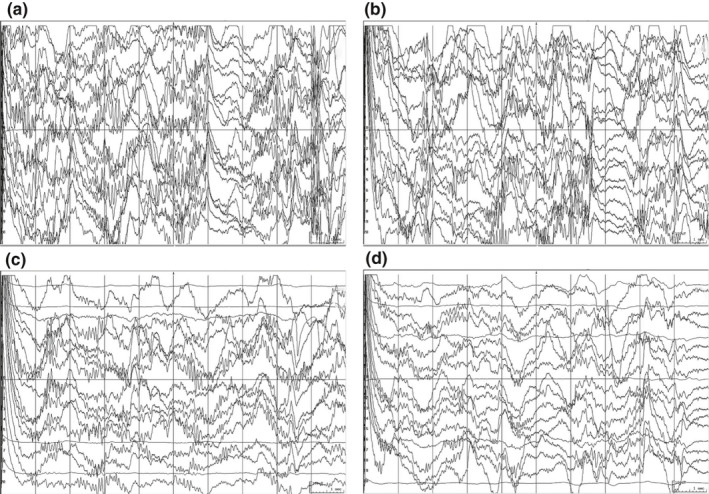
The dynamic EEGs pre‐ and postoperation of "hollow" and multiple subpial resections. (a) EEG of the super frontal gyrus before the operation; (b) the EEG after the first operation; (c) the EEG of the super frontal gyrus after the second "hollow" and resection; (d) normal EEG after the surgery

A total of 34 patients were randomly grouped before surgery, the first 15 patients (Patient number 1–15) were regarded as the control group which was not informed of the fMRI results and underwent operation by intraoperative EEG neuronavigation only. The rest (Patient number 16–34) are the experimental group that was under notification of the fMRI results, and the neurologists tried to avoid or reduce the damage of the activated area based on task‐state fMRI and intraoperative EEG in the experimental group, to achieve individualized precise protection. The hollowing and multiple subpial transections were performed in all 34 patients. In the hollowing craniotomy, exploring electrodes were carried out in the cortex to find and digitally mark the epileptic focus by intraoperative EEG. The hollowing was performed in the marked lesion regions about 0.5 × 0.5 × 0.8 cm under general anesthesia, to resection the most obvious areas of spike‐wave distribution by neuronavigation. The specific method multiple subpial transections was to poke to a hole with a needle in the avascular area of gyrus, and the leptomeninges was incised under a microscope, then inserted cutting knife along the hole to the contralateral limbic. The cutting knife emerged from the cortex without penetrating leptomeninges and always kept perpendicular with leptomeninges. The interval of every cut was 5–8 mm, with its depth of 5 mm. The lateral fibers of the cortex were incised until the spike‐wave disappeared or returned to normal. The process of surgery treatment is shown in Figures 2 and 3.

### 2.7 Statistical analysis

The fMRI data were analyzed using SPM8 software described above. Statistical analysis was carried out using the software SPSS 20.0. The measurement data were expressed as mean ± standard deviation, and the comparison between the two groups was analyzed by *t* test. The counting data were expressed as percentage or ratio, and the comparison between groups was analyzed by the chi‐square test. Single‐factor analysis of variance was done to compare the data obtained. Significant differences were considered as *p* < .05.

## 3 RESULTS

### 3.1 Comparison of general information

The 34 patients (average age 29.7 years, range 18–59) were accomplished surgery and experiments. There was no difference in age, gender, handedness, and handedness scores of the two groups (*p* > .05), which were comparable. The comparison of general information in the two groups is shown in Table 2.

**TABLE 2:** The comparison of general information in groups

Group	Number of cases	Age	Gender (F/M)	Handedness(R/L/B)	Handedness score
		(yr)			
The control group	15	29.9 ± 7.2	10/5	14/0/1	69.7 ± 12.9
The experimental group	19	29.6 ± 11.3	11/8	14/1/4	58.4 ± 30.9
*t/x^2^*		0.089	0.273	2.362	1.392
*p*		.929	.601	.307	.176

### 3.2 Ontoanalysis

The distribution of effective activation regions in 34 patients (intra‐object analysis) is shown in Table 3. The brain regions related to Chinese function activated by different tasks were remarkably distinct and mainly concentrated in temporal lobe and frontal lobe. The activation region of AN task mainly was in right superior temporal gyrus, bilateral middle temporal gyrus, and right middle frontal gyrus. The activation region of PN task mainly was in bilateral superior temporal gyrus and right middle frontal gyrus. The activation region of FF task mainly was in right superior temporal gyrus and left middle temporal gyrus. The activation region of SF task mainly was in bilateral superior temporal gyrus, bilateral superior frontal gyrus, and left middle frontal gyrus. The activation region of VFL task mainly was in right superior temporal gyrus, left middle temporal gyrus, and bilateral middle frontal gyrus. The activation region of VFC task mainly was in left middle temporal gyrus, right inferior frontal gyrus, and bilateral middle frontal gyrus. Meanwhile, activation signals of fusiform gyrus, parahippocampal gyrus, hippocampus, and precentral gyrus were generally low or even could not be detected.

**TABLE 3:** Global maxima activation (GMA) of 34 patients‐‐‐first level (intra‐object analysis)

Contrast	AN	PN	FF	SF	VFL	VFC
STG_L	8	11	5	10	7	5
STG_R	18	12	8	9	10	7
ITG_L	5	4	3	3	5	2
ITG_R	4	1	1	3	2	2
MTG_L	11	6	7	3	10	11
MTG_R	9	3	5	7	5	6
IFG_L	7	8	4	4	4	6
IFG_R	8	6	3	4	5	9
MFG_L	8	6	6	8	9	9
MFG_R	14	9	3	5	10	9
SFG_L	3	7	5	8	3	7
SFG_R	4	4	3	7	4	5
FuG_L	0	4	0	0	0	0
FuG_R	1	2	3	1	1	2
PHG_L	1	1	1	0	0	1
PHG_R	1	2	2	0	1	0
HC_L	0	1	1	1	1	1
HC_R	1	2	0	0	0	0
PG_L	0	3	0	1	2	1
PG_R	0	0	2	0	1	4

Note

Main effects from the different language paradigms and their specified contrasts, indicating the numbers of subjects with activations greater than a threshold of T >2.5, with extent >10 voxels.

Abbreviations: FuG, fusiform gyrus; HC, hippocampus; IFG, inferior frontal gyrus; ITG, inferior temporal gyrus; L, left; MFG, middle frontal gyrus; MTG, middle temporal gyrus; PG, Precentral gyrus; PHG, parahippocampal gyrus; R, right; SFG, superior frontal gyrus; STG, superior temporal gyrus.

### 3.3 Group analysis

The differences in the above distribution in 3.2 were analyzed with full factors, and the result of group analysis is shown in Table 4. The main effect regions of AN and PN task were in right superior temporal gyrus. The main effect regions of FF and VFC task were in right middle temporal gyrus. The main effect region of SF task was in left superior temporal gyrus. The main effect region of VFL task was in right middle frontal gyrus.

**TABLE 4:** Global maxima activation (GMA) of 34 patients: second level (full factors)

SP	NV	MNI			T
AN	1,448	−48	20	−16	4.738
	1,763	60	−14	−4	4.8881
	264	58	−4	42	3.6492
PN	13,784	30	−92	6	8.307
	165	−50	18	−14	3.7452
	167	−20	−22	−14	3.2592
	67	−60	−14	−2	2.8515
	236	−52	−8	52	3.3754
	200	64	−22	0	3.4401
	57	50	−8	30	2.9893
	85	58	8	−10	3.8413
	34	−2	−46	6	2.9376
	37	58	−6	46	3.0654
FF	294	26	−62	−30	3.2636
	107	66	−16	−7	3.3351
	35	−14	−60	−24	2.8126
	1,168	−28	0	30	3.4196
	15	−36	−28	−14	2.7872
	13	−32	−40	22	2.5505
SF	552	−60	−12	4	3.551
	19	18	−64	−26	2.7577
VFL	1,305	38	−62	−32	5.8995
	751	−34	−48	38	5.3561
	3,329	−52	2	50	6.7791
	80	60	0	36	3.2011
	559	−4	6	60	5.6979
VFC	154	38	−68	−32	3.7112
	1,304	−44	12	24	4.7937
	12	−40	−10	38	2.7721
	248	−30	32	0	3.5633
	42	30	28	12	3.0258
	49	−30	−48	6	3.194
	229	−52	−4	52	4.9794

Note

MNI, peak MNI coordinate; NV, number of voxels; SP, stimuli paradigm; T, peak intensity.

### 3.4 language ability

As shown in Table 5, the ABC assessment score of the control group 6 months after surgery was significantly lower than that 1 week before surgery (*p* < .05), while the ABC assessment score of the experimental group was 503.1 ± 76.7 6 months after surgery, which had no significant difference with that 1 week before surgery (*p* > .05). Besides, although there was no difference in the ABC assessment score of the two groups 1 week before surgery and 6 months after surgery (*p* > .05), the score of the experimental group was higher than that of the control group.

**TABLE 5:** The comparison of language ability in groups

Group	Number of cases	The ABC assessment score	
		1 week before surgery	6 months after surgery
The control group	15	553.7 ± 82.1	481.5 ± 63.4
The experimental group	19	558.5 ± 104.5	503.1 ± 76.7
*t*		0.145	0.878
*p*		.885	.368

## 4 DISCUSSION

Epilepsy is one of the most common diseases of the central nervous system, with over 50 million patients worldwide. There are about 9.84 million epilepsy patients in China and about 236,000 in Sichuan province. The incidence rate is higher in rural areas, and up to 30% are intractable epilepsy (Song et al., 2017). Epilepsy not only causes harm to the physical and mental health of patients but also brings a heavy economic burden to the family and society.

Epilepsy, which is difficult to treat by drugs, preferentially occurs in the temporal lobe (Perucca & Tomson, 2011). The main treatment is surgery, such as anterior temporal lobectomy and modified hollowing at present. And it is worth noting that the temporal lobe, amygdala, and hippocampus structures play an important role in cognitive functions, and the damage of them would result in the corresponding language, memory, and other cognitive function lesions (Campo et al., 2013; Hauptman et al., 2012; Noppeney et al., 2005). Besides, pathological changes always are inconsistent with the focus (Clarke et al., 1996). Therefore, most of patients would appear with different levels of language dysfunction after surgery, and it is urgently needed to prevent these complications in clinical.

The outcome of surgery mainly depends on the accurate localization and excision of epileptogenic focus (Ren et al., 2015). Traditional intraoperative EEG could roughly locate the epileptogenic focus because of its lower special resolution, and it could not effectively mark the functional language regions of patients in the brain (Blumer et al., 1998). In recent years, the emergence of BOLD‐fMRI technology, whose signals are the hemodynamic response of a large number of nerve cells, provides the possibility of accurate localization of epileptogenic focus. Based on its advantages of noninvasive, high sensitivity and resolution, BOLD‐fMRI technology has been used to study epileptic brain networks. In our paper, BOLD‐fMRI and intraoperative EEG were combined to realize the complementary.

Our results showed that different brain regions were activated by different tasks, and the activated regions of the same task were different in each individual, and the regions of different tasks overlapped with each other (Table 3). The distribution of significantly activated regions of the same task was studied with group analysis with full factors: The main effect regions of AN and PN task were in right superior temporal gyrus. The main effect regions of FF and VFC task were in right middle temporal gyrus. The main effect region of SF task was in left superior temporal gyrus. The main effect region of VFL task was in the right middle frontal gyrus (Table 4). The distribution of significantly activated regions mainly was concentrated in the temporal lobe and frontal lobe, which was consistent with previous research (Duncan, 2010). The results of group analysis was different from those of ontoanalysis, which could not represent the distribution of individuals and was not suitable for guiding surgery. And our experience has shown that the surgical plans based on previous group analysis will damage more functional areas, which may be an important reason of side effect in routine epilepsy surgery. The usual practice is that the regions with the highest degree of activation should be protected firstly. So in the best avoiding scheme, the hollowing and transections of nerve fiber should be performed in the nonactivated regions beyond the intersection (Binder et al., 2011; Mahvash et al., 2014), and the activated regions should be preserved to the greatest extent. Among different individuals, the distribution of significantly activated regions of the same task was different, so it is necessary to combine fMRI and intraoperative EEG to reduce the damage of functional language regions and develop personalized programs.

Unlike Western languages, most Chinese characters are pictographic characters (80%). They are the most representative graphic characters and have a complex block structure composed of multiple strokes. Therefore, the process of Chinese characters also requires more brain regions to participate in (Li et al., 2018). According to the particularity of Chinese, our experiment was pertinently designed to assess the functional language regions related to viewing, listening, and speaking. This paper improved the task of semantic association by exploring the relevant brain regions where the circuit for language was located from the perspective of phonetic and semantic elements (Li et al., 2018; Limotai & Mirsattari, 2012; Tie et al., 2009). Our results showed that the activated regions of the same task were not isolated specific points, but multiple brain regions in piece‐like distribution. Besides, the regions were in a relatively concentrated range (Perucca & Tomson, 2011), which performed the compound function of multiple brain regions (Table 3). This also proved the process of Chinese characters was the coordination of multiple brain regions, which was consistent with the results of other studies (Li et al., 2018). The ABC assessment score of the control group 6 months after surgery was significantly lower than that 1 week before surgery (*p* < .05), while there was no significant difference in the experimental group, and the score of the experimental group was higher than that of the control group. This means that the hollowing and modified multiple subpial transections were successfully performed based on task‐state fMRI and intraoperative EEG, and the language function of the experiment group was better protected.

## 5 CONCLUSION

Language functional task‐state brain magnetic resonance imaging is a new way to explore the language damage caused by epilepsy. In the surgical treatment of epilepsy, a personalized surgical plan, based on task‐state fMRI and intraoperative EEG, can be developed according to the difference of activation areas to protect the language function and improve the quality of life in postoperative patients.

## CONFLICT OF INTEREST

There are no conflicts of interest.

## AUTHOR CONTRIBUTION

Wang Peng and Jiang Rui conceived the ideas; Du Feizhou and Li Jianhao collected the data;Wang Peng and Du Feizhou analyzed the data; Yu Hongmei and Tang Chencheng led the writing.

### Peer Review

The peer review history for this article is available at https://publons.com/publon/10.1002/brb3.1979.

## Data Availability

The data that support the findings of this study are available from the corresponding author upon reasonable request.
